# Decentralized Smart-Hive IoT Architecture and Open Field Telemetry Dataset

**DOI:** 10.3390/s26206560

**Published:** 2026-10-16

**Authors:** Igor Kurdin, Marcin Bator, Arkadiusz Orłowski

**Affiliations:** 1 Department of Artificial Intelligence, Faculty of Computer Science and Engineering, Warsaw University of Life Sciences—SGGW, Nowoursynowska 159, 02-776 Warsaw, Poland; igor_kurdin@sggw.edu.pl (I.K.); arkadiusz_orlowski@sggw.edu.pl (A.O.); 2 Faculty of Cybernetics, Military University of Technology, 00-908 Warsaw, Poland; arkadiusz.orlowski@wat.edu.pl

**Keywords:** smart hives, autonomous IoT architecture, solar-powered monitoring, GSM telemetry, commercial pollination, open dataset, precision beekeeping, smart agriculture

## Abstract

Open smart-hive datasets collected under commercial pollination conditions remain scarce and often lack homogeneous timestamps and synchronized event metadata. We present the decentralized AmoHive monitoring architecture and an open hourly IoT telemetry dataset collected from nine structurally identical solar-powered hives from 1 May to 31 August 2024 in the Kyiv region of Ukraine. Each hive operated as an autonomous sensing and GSM communication node without a local concentrator, allowing four instrumented colonies to be relocated to two sunflower fields without reconfiguring shared infrastructure. System operation was evaluated through temporal alignment, node-level coverage, missing-data runs, channel completeness, consistency of co-located external sensors, and a limited reconstruction check. All nodes produced records compatible with the common hourly grid. Observed coverage ranged from 76.6% to 95.8%, and missing-data runs were predominantly one to two hours. Co-located external-temperature channels tracked shared conditions consistently, whereas relative-humidity channels showed larger unit-specific differences. The released resource contains approximately 26.5 thousand hourly multichannel records, paired RAW and CLEAN layers, campaign metadata, a timestamped beekeeper-event workbook with an accompanying embedding script, and value-level provenance. The scientific contribution is a system-level field evaluation of a relocatable per-hive IoT topology coupled with a traceable dataset that connects physical deployment, node-specific data quality, and reproducible preprocessing.

## 1. Introduction

### 1.1. Precision Beekeeping and the Need for Reproducible Telemetry Datasets

Precision beekeeping is increasingly becoming part of the broader smart-farming paradigm in which management decisions are informed by continuous sensor-based monitoring [1,2,3]. Modern smart-hive systems can record colony weight, internal and external temperature, relative humidity, acoustic signals, and other environmental or technical variables using distributed IoT infrastructure [4,5,6]. Such systems are expected to support early stress detection, operational planning, colony-health monitoring, and long-term analysis of colony–environment interactions.

The use of smart-hive data is increasingly extending from retrospective monitoring to forecasting and automated detection of abnormal colony states. Recent studies have used weight, temperature, humidity, and acoustic time series for anomaly detection, daily weight estimation, colony-state classification, and decision support [7,8,9]. The same hourly AmoHive monitoring architecture and a comparable RAW-to-CLEAN processing approach were also used to develop an early-warning detector for the rapidly developing phenomenon of honey robbing [10]. These applications depend on regular and well-documented time series because model outputs can be affected by missing records, timestamp irregularities, and unreported data reconstruction [2].

Hive weight is a particularly informative monitoring variable because its daily dynamics reflect several simultaneous processes, including forager departure and return, resource inflow, food consumption, respiration, and moisture exchange [11]. Regular weight time series can also be used to estimate activity-related colony parameters and resource collection patterns [12]. Internal and external temperature and relative humidity provide additional environmental and microclimatic context. External temperature and solar conditions can substantially affect food collection and hive-weight changes, whereas the relationship between internal and external temperature provides information about the thermal state of the colony [13,14].

At the same time, the practical deployment of smart-hive infrastructure under field conditions introduces substantial methodological difficulties. Unlike laboratory or short-term experimental datasets, real-world IoT telemetry is inherently heterogeneous and affected by missing data, irregular timestamps, variable sampling intervals, transmission interruptions, duplicate observations, sensor drift, calibration artifacts, energy limitations, and management-related signal disturbances [15,16]. Consequently, raw telemetry cannot usually be used directly for statistical comparison, machine-learning workflows, or cross-hive interpretation without explicit preprocessing.

Several longitudinal smart-hive datasets containing weight, temperature, humidity, acoustic, or phenotypic information have recently been released [15,16,17]. These resources have demonstrated the value of standardized sensor records for comparative analysis, benchmarking, and model development. For example, existing resources include long-term weight and microclimate records from multiple colonies and multi-sensor datasets accompanied by expert-derived phenotypic observations.

However, available datasets differ substantially in temporal resolution, monitoring duration, number of colonies, sensor modalities, event annotation, preprocessing transparency, and the availability of RAW and processed records. Openly available datasets collected during real commercial pollination campaigns remain limited. Beyond the longitudinal resources noted above [15,17], published resources also include a winter-feeding dataset [18] and an event-oriented smart-hive study of honey robbing [19]. Few resources combine an explicit pollination-campaign structure with synchronized management events, transparent preprocessing, and per-value provenance for reconstructed observations [16].

The challenge becomes especially important in commercial pollination campaigns, where colonies are transported between locations and monitored under operational rather than laboratory conditions [20,21,22]. In such settings, telemetry interpretation requires synchronized timestamps, transparent event logs, and reproducible quality-control rules. Without these components, downstream analyses risk conflating biological variability with technical artifacts.

The engineering problem is therefore not limited to installing sensors in a hive. A monitoring system for commercial pollination must remain operational when selected colonies are moved away from the home apiary, avoid dependence on cables or shared local communication infrastructure at temporary field sites, and still produce comparable, traceable multichannel records across locations. Existing monitoring approaches reviewed in Section 2 range from independent cellular units to sensor networks using local gateways or concentrators, while the available datasets differ in whether RAW records, processing rules, event metadata, and reconstruction provenance are released. The present work links these two requirements: a relocatable per-hive monitoring architecture and a documented path from field telemetry to reusable data.

We address this gap by documenting the decentralized AmoHive architecture, demonstrating its operation during a commercial pollination campaign, and releasing the resulting telemetry with the workflow used to produce the CLEAN records.

### 1.2. Scope and Contributions

This article concerns the AmoHive monitoring architecture, its field deployment, and the resulting open data resource. The pollination campaign supplies the operational setting and event structure; biological interpretation of colony dynamics and the effect of the attractant are not analyzed here. The RAW-to-CLEAN procedure is reported only to make the released processed records reproducible.

The first contribution is a field demonstration of a decentralized smart-hive architecture in which every AmoHive unit is a complete IoT node with integrated sensing, solar power and battery buffering, and independent GSM communication. Without a local concentrator, individual monitored hives can be added, removed, or relocated without reconfiguring shared communication infrastructure.

The second contribution is an open multichannel telemetry dataset from nine structurally identical hives monitored from 1 May to 31 August 2024, containing approximately hourly RAW telemetry, regularized hourly CLEAN records, and documented field and intervention events.

The third contribution is the exact reference workflow used to derive the CLEAN records from the RAW telemetry, including value-level provenance that distinguishes observed from reconstructed values.

The present study builds on three earlier outputs but addresses a different analytical level. Reference [19] introduced the design and operation of the autonomous AmoHive unit and applied telemetry from that platform to monitoring and forecasting honey-robbing events. Reference [23] described the pollination dataset as a Data Note, while reference [24] provides the public data repository and accompanying materials. The present article examines the system formed by multiple autonomous hives: its decentralized topology, independent communication and power supply at each node, reconfiguration through relocation of selected hives, and operational data continuity during a commercial pollination deployment. Table 1 summarizes both the scope of the earlier outputs and the analytical approach used in the present study to extend each of them.

## 2. Related Monitoring Systems and Datasets

### 2.1. Monitoring Approaches and Temporal Organization

The development of continuous monitoring of honey bee colonies for both scientific research and practical beekeeping was anticipated before the widespread adoption of modern IoT systems. Meikle and Holst considered continuous monitoring as colony-level data collection performed hourly or more frequently and, importantly, emphasized that monitoring hive weight or forager traffic before crop pollination could help assess colony condition, whereas data collected during pollination could provide a record of quality control [25]. They also highlighted the synergy of simultaneously measuring multiple variables, where one type of data can validate or augment information obtained from another.

A recent study proposed that modern approaches to hive monitoring in precision apiculture fall into two main categories: computer vision-based systems and sensor-based time-series monitoring [7]. The first approach provides more direct observation of individual bees, movement at the hive entrance, behavioral patterns, or visible anomalies. Its advantage lies in visual detail, but practical autonomous implementation of such monitoring under field conditions requires substantially greater energy, computational, and communication resources. The second approach does not directly record the behavior of individual bees, but allows long-term, autonomous, and non-invasive monitoring of the colony as an integrated biological system through regular time series of hive weight, temperature, humidity, and technical parameters.

Comparison of sensor-based systems requires a distinction among the sensor-acquisition interval, the interval at which measurements are stored as records, the interval at which records are transmitted to a remote infrastructure, and the temporal resolution ultimately used for analysis. These quantities are not necessarily equivalent. A system may acquire measurements every few seconds, transmit them in batches, and subsequently analyze hourly aggregates. Conversely, a low-duty-cycle node may wake at longer intervals, acquire one multichannel record, transmit it, and then return to sleep. Therefore, terms such as “continuous” and “real time” do not by themselves define the temporal structure of the resulting dataset.

Published non-invasive sensor systems employ substantially different temporal strategies depending on the monitored phenomenon, power architecture, communication technology, and intended analytical use. Table 2 summarizes representative primary studies in which acquisition or logging intervals, analytical treatment, and transmission or storage strategies are described with sufficient detail.

The monitoring interval depends on the phenomenon being studied. Minute-level measurements are appropriate for the timing or shape of short within-day transitions, such as initial forager departure, return activity, or rapid changes in hive weight. Less frequent measurements may be sufficient for long-term colony-weight trends, daily resource balance, persistent microclimatic changes, and seasonal comparisons.

Under field conditions, very frequent measurement increases temporal resolution but also raises energy consumption, data volume, radio activity, and dependence on solar power, battery buffering, and communication stability. Sparse collection saves energy but may miss within-day weight dynamics, microclimatic transitions, and short-term signals.

### 2.2. Positioning of the AmoHive Deployment

Sensor time series can complement visual, acoustic, molecular, ecological, and beekeeper-derived evidence in early-warning applications [30,31]. In earlier AmoHive work, multi-hive time series supported early detection of honey robbing [10], illustrating the downstream value of consistent longitudinal telemetry.

In the present dataset, the multi-sensor monitoring logic is implemented through hourly hive-weight telemetry, where hive weight acts as an indirect but biologically interpretable colony-level proxy for foraging activity, resource inflow, food consumption, moisture exchange, and operational changes [11,12,25]. The dataset presented in this paper represents a practical field implementation of this logic during a commercial sunflower pollination campaign.

Hourly hive-weight monitoring before relocation, together with pre-campaign inspections, supported comparison of colony condition and formation of groups with comparable strength. During pollination, the weight records captured broad day-scale changes and operational events, while synchronized internal and external temperature and humidity provided microclimatic context.

The hourly interval balances biological informativeness with the energy and communication constraints of autonomous field operation. It resolves broad within-day and seasonal weight patterns, although not the exact timing of rapid sub-hourly transitions; earlier electronic-hive studies used hourly or more frequent records for related purposes [19,32,33].

Reusable field telemetry requires standardized timestamps, explicit metadata, documented handling of missing observations, and a distinction between observed and reconstructed values [1,3,15,16,17]. The released data combine season-long records with a timestamped event workbook that can be synchronized to the hourly grid and with value-level provenance.

Bees detect biologically relevant electric fields, and controlled studies have reported biochemical or behavioral responses to artificial electric, magnetic, and radio-frequency fields [34,35,36,37,38,39]. Those exposure conditions differ from AmoHive operation, and electromagnetic fields were not measured in this deployment.

During each hourly cycle, the AmoHive electronics and GSM transmitter remain active only for the few minutes needed to acquire and transmit a record [19]. This low-duty-cycle design primarily reduces energy demand and also limits active radio-operation time near the colony; no biological effect is inferred.

## 3. Materials and Methods

### 3.1. Smart-Hive Monitoring System

**Figure 1:**
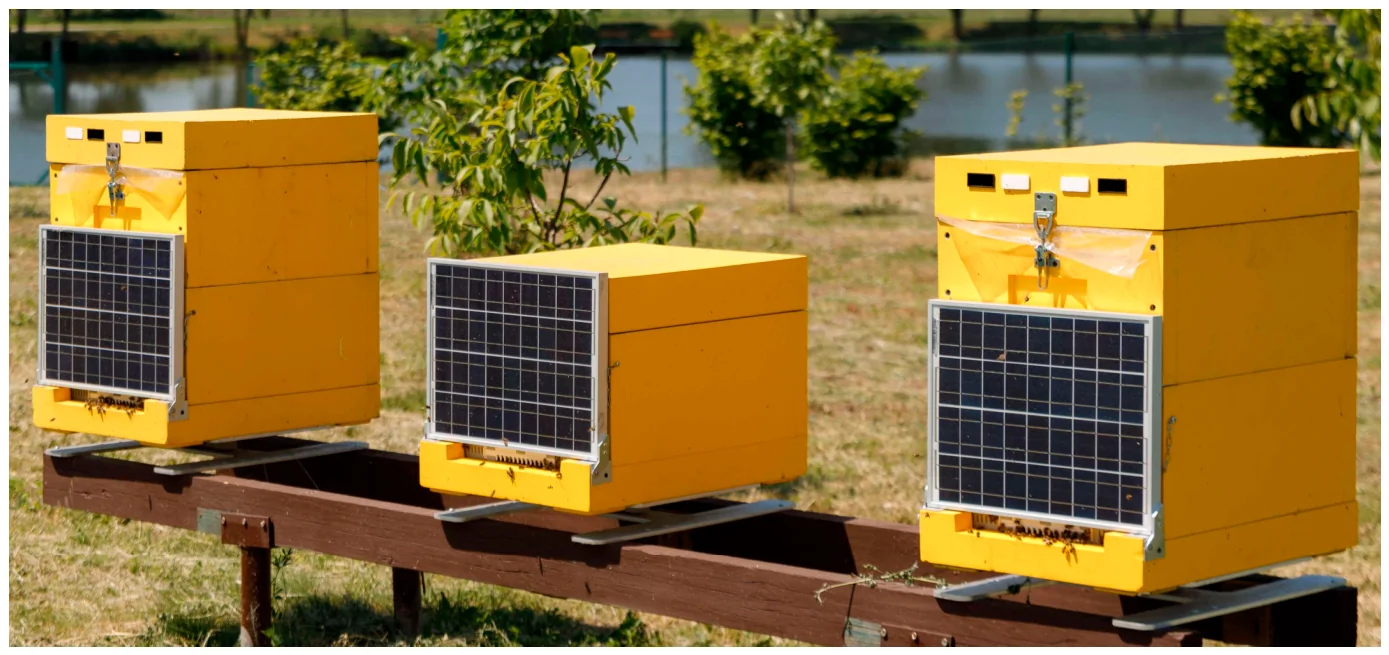
AmoHive smart-hive monitoring system used for field telemetry collection. The system combines hive-weight measurement, internal and external temperature and humidity sensing, GSM-based data transmission, and solar-powered autonomous operation.

The dataset was collected using AmoHive smart hives, an autonomous IoT platform designed for long-term non-invasive monitoring of honey bee colonies under field conditions. In this paper, smart refers to autonomous networked sensing, telemetry, and remote monitoring; the reported system does not incorporate an artificial-intelligence or machine-learning inference model. The field implementation is shown in Figure 1.

The system uses a decentralized per-hive topology: every monitored hive contains the complete sensing, power-management, timing, and cellular-communication chain and communicates directly with the remote server. No shared local gateway or apiary-level concentrator is required. Consequently, failure or relocation of one unit does not interrupt telemetry from the remaining hives, and the monitored set can be reconfigured by moving individual hives. Multiple co-located autonomous units also provide parallel measurements of external conditions that can be used for cross-checking. These parallel records are not assumed to be interchangeable or equivalent to measurements from a calibrated weather station.

**Figure 2:**
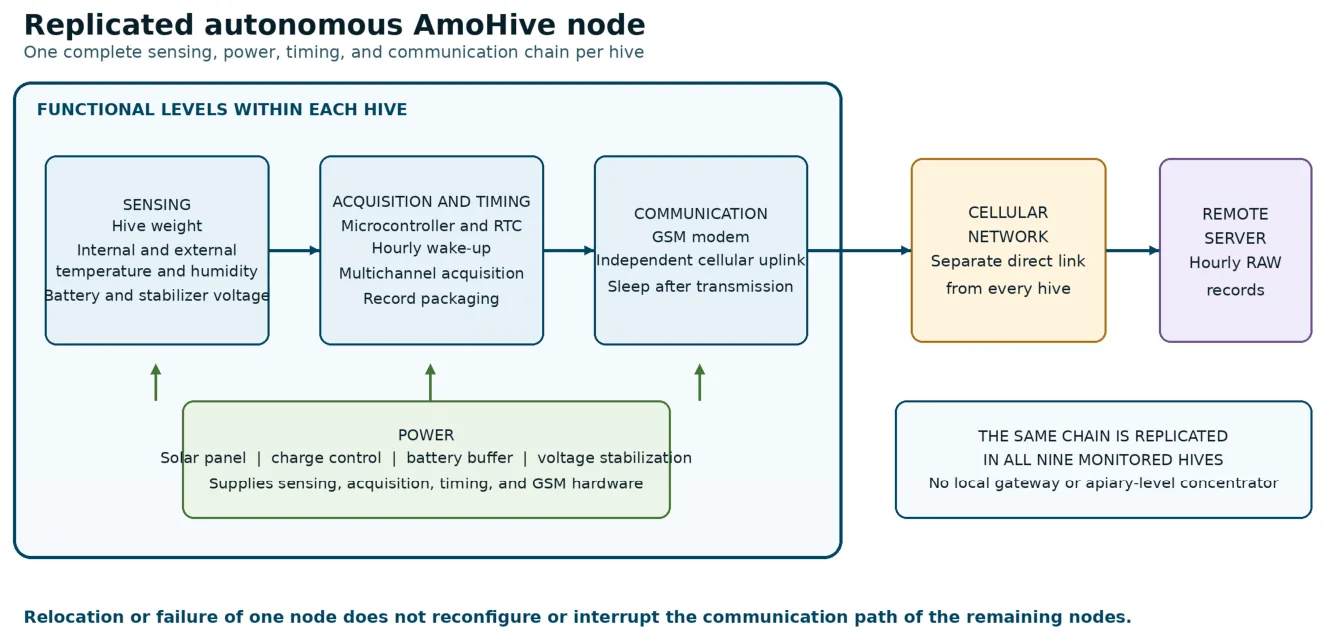
Functional architecture of the decentralized AmoHive monitoring system. Each hive contains a complete sensing, power, timing, and GSM communication chain and transmits directly through the cellular network to the remote server.

Figure 2 separates the architecture into four functional levels within each hive. The sensing level acquires hive weight, internal and external temperature and humidity, and power-system voltages. The acquisition and timing level wakes the node, reads the channels, and packages one hourly record. The power level supplies the complete node from the solar panel and battery buffer, while the communication level sends the record through the hive’s own GSM modem to the remote server. This chain is replicated in all nine monitored hives; no apiary-level device aggregates or relays their traffic.

Each hive carries its own power and GSM hardware, and cellular coverage is required for transmission. Compared with a shared-gateway architecture, this duplicates communication, power-management, and buffering components at every hive and distributes maintenance across the apiary. It may also require a separate SIM or data service for each node, depending on the cellular plan. Each unit recorded:

- Hive weight,
- Internal temperature,
- External temperature,
- Internal relative humidity,
- External relative humidity,
- Processor temperature,
- Stabilized solar-energy proxy.

The monitoring units operated in a low-duty-cycle mode with a nominal telemetry interval of approximately 60 min. During each scheduled cycle, the controller activated the sensing subsystem, acquired one complete multichannel record, transmitted it through the GSM module, and returned to sleep mode [19]. The active phase lasted only a few minutes. Both acquisition and transmission therefore occurred approximately once per hour; the RAW files contain one record per scheduled cycle rather than higher-frequency measurements aggregated after collection.

The main hardware components of the AmoHive monitoring unit are summarized in Table 3.

The system was powered using a solar panel combined with rechargeable battery buffering, enabling continuous season-long telemetry without external power infrastructure [19]. The combination of solar power, battery buffering, and low-duty-cycle operation was intended to reduce energy demand and support autonomous field deployment while limiting the duration of active electronic and radio operation near the colony. The variable e_uakuv is the electrical voltage measured after the solar-panel stabilizer (V). It is retained as a technical indicator of the node’s solar-powered electrical system, not as a calibrated measurement of solar irradiance or environmental light intensity.

All monitored hives followed the Langstroth standard and used the same hive-body and electronics architecture, reducing technical differences among monitoring units.

### 3.2. Study Design and Campaign Structure

**Figure 3:**
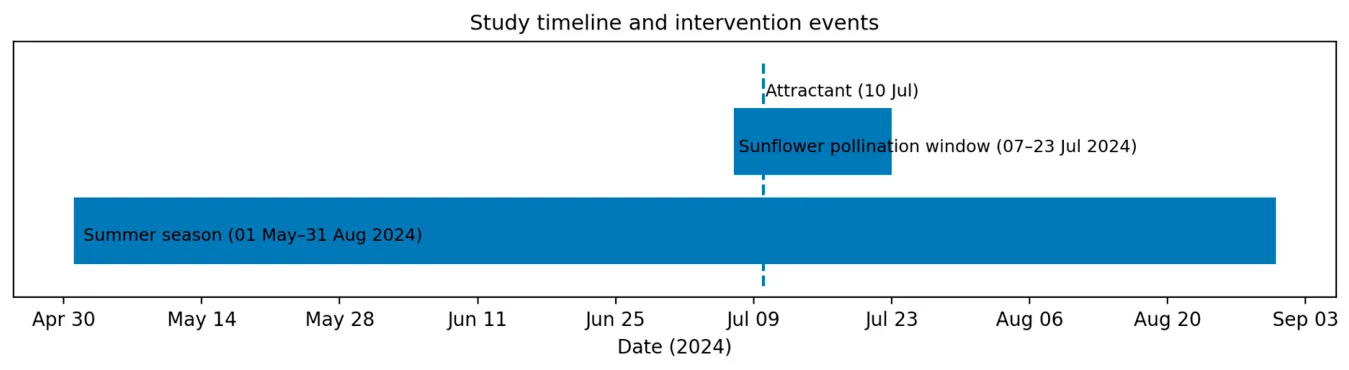
Study timeline for the 2024 monitoring period. The figure shows the full observation window from 1 May to 31 August 2024, including the commercial sunflower pollination campaign, hive relocation events, and the attractant application date.

The monitoring period covered 1 May to 31 August 2024 and included nine monitored colonies located in the Kyiv region of Ukraine. The campaign timeline is summarized in Figure 3.

The observation period included a commercial sunflower pollination campaign conducted between 7 and 23 July 2024, during which selected colonies were relocated from the stationary apiary to sunflower fields.

The campaign was treated as an operational field deployment of the monitoring architecture rather than as a randomized trial of biological treatment efficacy. The autonomous hive node was the unit used to assess system operation; campaign-group assignments and management events were retained as deployment metadata. Four monitored colonies were relocated to two sunflower fields, while five remained at the stationary apiary.

The campaign structure consisted of three observational groups:

1. Field with attractant application,
2. Field without attractant,
3. Stationary apiary controls.

The field-with-attractant group comprised hives 027 and 430. The field-without-attractant group comprised hives 591 and 985. The stationary apiary control group comprised hives 224, 286, 448, 841, and 883.

An ecological attractant was applied on 10 July 2024 using an agricultural UAV. Campaign dates, hive relocation, group allocation, and attractant application are documented in the associated Data Note [23]; timestamped beekeeper events are supplied in a separate workbook.

### 3.3. Dataset Structure

Raw telemetry files are released per hive as timestamped delimited tables. Each record includes local timestamp information together with all sensor measurements.

All sensor channels are acquired and transmitted together in one multichannel record. The absence of a RAW record therefore represents the loss of the complete scheduled multichannel observation. Missing or technically implausible values within a received record are handled separately by channel-specific quality control and provenance fields in the CLEAN layer.

The released dataset consists of two major layers:

1. Raw telemetry,
2. Cleaned hourly telemetry.

The cleaned telemetry layer contains:

- Regularized hourly timestamps,
- Reconstructed values,
- Provenance labels describing the origin of each value.

The dataset repository additionally contains the processing scripts, a formal method specification, a variable and provenance-label dictionary, the beekeeper event workbook, software dependencies, integrity checksums, and citation and licensing metadata [24].

### 3.4. Reference RAW-to-CLEAN Workflow

**Figure 4:**
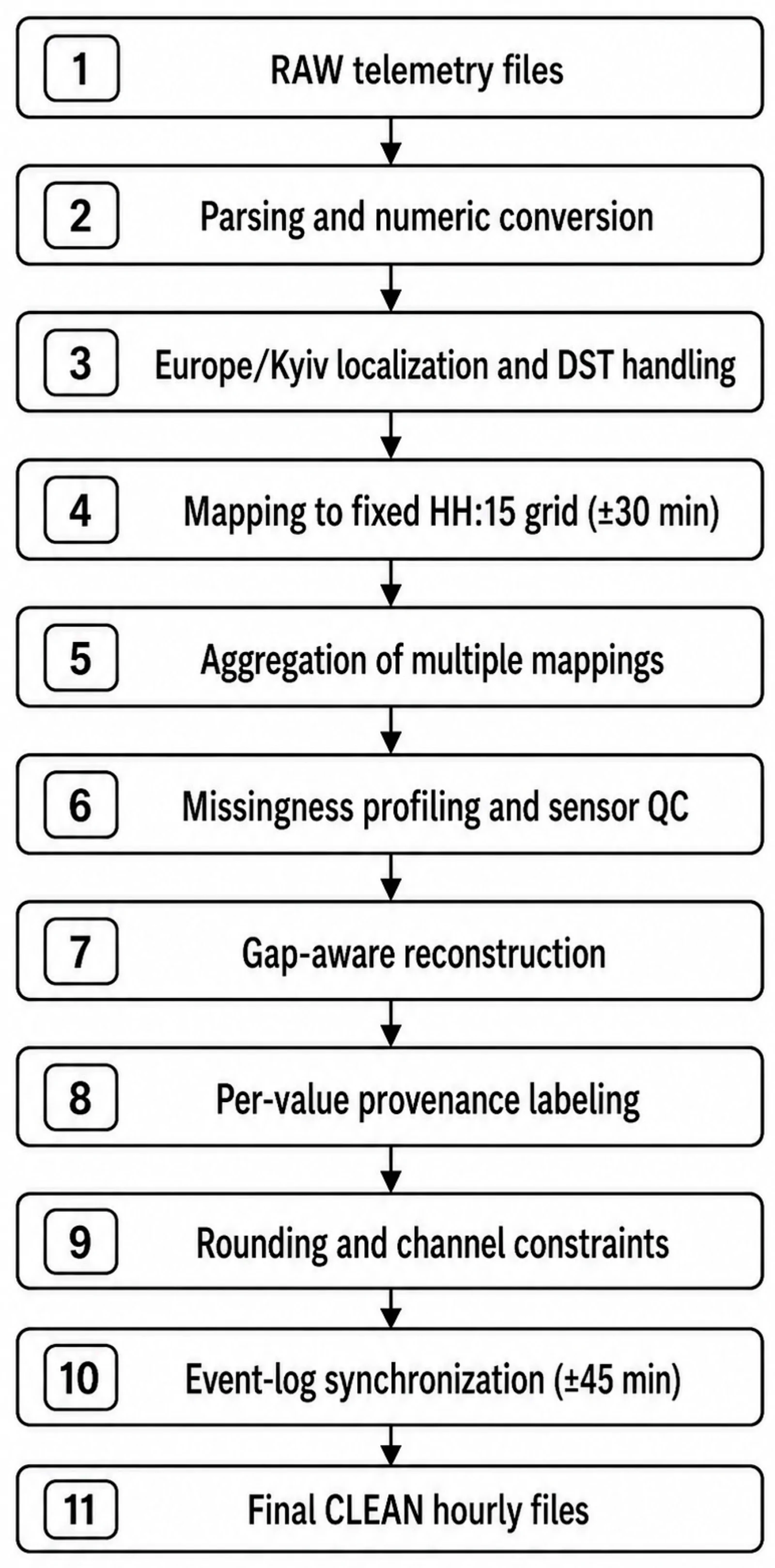
Reference RAW-to-CLEAN processing workflow used to produce the released hourly CLEAN files from the original smart-hive telemetry.

The CLEAN layer was produced from the original device exports using the deterministic reference workflow summarized in Figure 4.

The workflow comprised nine deterministic stages: (1) parsing RAW timestamps and sensor values; (2) interpreting timestamps in the Europe/Kyiv time zone with daylight-saving-time handling; (3) mapping observations to the fixed HH:15 hourly grid; (4) averaging duplicate observations assigned to the same grid point; (5) identifying missing and technically implausible values; (6) reconstructing gaps using channel- and gap-length-specific rules; (7) assigning a provenance label to every reconstructed value; (8) applying channel-specific rounding and output constraints; and (9) enabling optional synchronization of beekeeper events with the regularized telemetry.

The grid minute was selected by evaluating candidate offsets from 00 to 59 and calculating, for each offset, the mean absolute circular displacement of all RAW timestamps from the nearest hourly grid point. HH:15 minimized this statistic for the pooled records from the nine nodes and was therefore used as the common hourly grid.

Let t_i denote a RAW timestamp and let G = {τ_k} be the hourly grid with τ_0 at minute 15. Each observation was assigned to the nearest grid point according to

$$k\left(i\right)=\underset{k}{\arg\;\min}\;\left|t_{i}-\tau_{k}\right|,\;\;\tau_{k}=\tau_{0}+k\cdot 1\;h,\;\;\left|t_{i}-\tau_{k\left(i\right)}\right|\leq 30\;\min.$$

Only mappings within 30 min were retained. An exact half-hour tie was assigned to the earlier grid point, matching the released implementation. When several RAW records mapped to one grid point, their channel values were averaged. Temperature values equal to 0 °C and relative-humidity values less than or equal to 0% or greater than or equal to 100% were treated as technical failures or saturation artifacts and set to missing before reconstruction. No statistical outlier-detection algorithm was applied: duplicate aggregation preceded this rule-based technical quality control, and quality control preceded gap reconstruction.

For humid1, humid2, weight, proctemp, and e_uakuv, gaps of at most five hourly points were reconstructed by linear interpolation. Longer gaps used the released regression procedure with cyclic hour-of-day and day-of-year predictors and a level offset; its coefficients were estimated from the variable-specific training window defined in the released processing code. For temp1 and temp2, gaps of at most six hourly points were interpolated; longer gaps used a per-hive, per-channel median diurnal profile. The interpolation between observed boundary values x(t_0) and x(t_1) was

$$\hat{x}\left(t\right)=x\left(t_{0}\right)+\frac{t-t_{0}}{t_{1}-t_{0}}\left[x\left(t_{1}\right)-x\left(t_{0}\right)\right].$$

A corresponding *_method field identifies each reconstructed value as linear-time, regress-time+offset, temp-rule-linear-time, temp-rule-diurnal-median, or, where applicable, temp-rule-edge-fill. A blank method field denotes a directly observed value retained after mapping and quality control. Thus, reconstruction can be excluded or stratified in downstream analyses.

Beekeeper events are supplied in pollination_events_2024.xlsx, with one worksheet per hive. Each record contains event_time_local, a categorical event_type, and a free-text manual_note. The released category list comprises inspection, frames_added_n, frames_extracted_n, super_added_n, super_extracted_n, sensor_maintenance, and sensor_artifact. The released embedding script assigns each event to the nearest HH:15 grid point within ±45 min and populates the event fields in a copy of the CLEAN table; the distributed CLEAN files contain the corresponding empty placeholder columns until this optional merge is performed. When several events map to the same grid point, event types are joined with ‘;’ and notes with ‘|’.

For example, the hive-027 worksheet records event_time_local = 2024-07-14 20:15, event_type = frames_extracted_n, and manual_note = ‘honey extraction, 5 frames, ~8.3 kg.’; the hive-448 worksheet records event_time_local = 2024-06-13 19:15, event_type = frames_added_n, and manual_note = ‘empty 5 frames’. Campaign dates, relocation, group assignment, and attractant application are retained separately as study-level metadata.

The released repository contains the RAW and CLEAN files, executable processing scripts, fixed parameter settings, the formal method specification, variable and provenance dictionaries, and the event workbook required to reproduce the transformation and optional event merge. The procedure is presented here as the reference method used to create the released CLEAN layer, not as an optimal reconstruction algorithm.

### 3.5. Technical Performance Measures

System performance was characterized using measures tied to the monitoring architecture and the released data rather than to biological outcomes. At the node level, these measures were the number of RAW records, median absolute timestamp displacement from the nearest HH:15 grid point, observed hourly coverage before reconstruction, and the median and maximum lengths of consecutive missing hive-weight records. Observed coverage was calculated as the number of mapped directly observed hourly records divided by the number of expected grid positions in the monitoring period. At the dataset level, the numbers and percentages of directly observed, reconstructed, and remaining missing values were calculated by hive and telemetry channel from the corresponding provenance fields.

For hive h, observed coverage before reconstruction was defined as

$$C_{h}=\frac{N_{\mathrm{obs},h}}{N_{\exp,h}}.$$

where N_obs,h is the number of mapped grid positions containing a directly observed record and N_exp,h is the number of expected hourly positions in the monitoring period. Consecutive empty positions were grouped into missing-data runs, whose lengths were expressed in hours.

Inter-hive consistency of the external sensors was assessed using simultaneous, directly observed values only; records carrying reconstruction labels were excluded. Pairwise Pearson correlation coefficients and median absolute pairwise differences were calculated for external temperature and relative humidity. Comparisons covered all 36 hive pairs while the nine units were co-located before relocation (1 May–6 July), and the 12 pairs within the three co-located groups during the pollination campaign (7–23 July): hives 027/430, hives 591/985, and the five stationary hives.

As a limited quality check of the released CLEAN layer, 2% of the observed hourly hive-weight records were randomly masked for each hive, reconstructed with the reference workflow, and compared with the original observations using mean absolute error (MAE) and root mean squared error (RMSE).

For the N masked observations, with original values x_i and reconstructed values $\hat{x}$_i, the two reported error measures were

$$\mathrm{MAE}=\frac{1}{N}\sum_{i=1}^{N}\left|x_{i}-{\hat{x}}_{i}\right|.$$

$$\mathrm{RMSE}=\sqrt{\frac{1}{N}\sum_{i=1}^{N}{\left(x_{i}-{\hat{x}}_{i}\right)}^{2}}.$$

This check evaluates isolated masked hive-weight observations only; it is not a comparative benchmark of alternative reconstruction algorithms.

## 4. Results

### 4.1. Temporal Consistency and Node-Level Data Availability

The nine autonomous nodes generated between 2257 and 2829 RAW records during the observation period. With the common HH:15 grid, node-level median absolute timestamp displacement ranged from 1 to 26 min and the maximum observed displacement was 30 min, the predefined mapping limit. These results show device-specific timing phases but no failure of temporal alignment across the decentralized system. Table 4 summarizes the node-level availability, timing, and gap measures.

Observed coverage ranged from 76.6% to 95.8% across nodes. The median missing-data run was one hour for eight hives and two hours for hive 448; the longest run ranged from 5 to 17 h. Thus, reduced completeness resulted mainly from repeated short interruptions rather than prolonged loss of an entire node. The marked differences among hives also show why availability must be reported per autonomous unit rather than only as an apiary-wide average.

The available telemetry does not contain device-diagnostic logs that would distinguish among RTC drift, wake-up scheduling, device configuration, and GSM or server latency as the cause of these node-specific phases. The offsets should therefore not be assigned to one mechanism. The ±30 min tolerance equals one half of the 60 min grid spacing and is the largest nearest-grid window that avoids overlap between adjacent hourly intervals, apart from the exact midpoint tie. A narrower tolerance would disproportionately discard records from nodes operating with a stable phase near 25–30 min and would reduce their apparent coverage. For this reason, per-node displacement statistics are reported, and analyses requiring sub-hour timing should use the released RAW timestamps rather than the regularized hourly grid.

### 4.2. Dataset-Wide Technical Summary

To characterize the released CLEAN layer without interpreting biological differences among hives or campaign groups, Table 5 reports pooled technical summaries for the seven telemetry channels. Directly observed, reconstructed, and remaining missing values were identified from the channel-specific provenance fields. Medians and interquartile ranges are reported because the pooled seasonal distributions are not assumed to be Gaussian.

The 0.0% minima in Table 5 do not represent retained direct sensor readings. They occur in 13 reconstructed values (eight for humid1 and five for humid2), all labelled regress-time+offset. After reconstruction, humidity outputs were constrained to the interval 0.0–99.9% and rounded to 0.1%; these 13 values therefore reached the lower output bound during post-processing.

The upper output bound of 99.9% accounts for 51.7% of non-missing humid1 values and 52.6% of non-missing humid2 values, with most of these values directly observed. The high pooled medians therefore reflect persistent upper-limit output in several sensor units rather than a calibrated estimate of absolute humidity. Together with the between-unit differences reported in Section 4.3, this indicates that the relative-humidity channels should be treated as unit-specific contextual signals. The available data do not distinguish among sensor response, enclosure effects, condensation, or calibration offset as the cause.

The e_uakuv values summarize the voltage recorded downstream of the solar-panel stabilizer. Their 12.03–14.25 V range characterizes operation of the node’s electrical system; it cannot be converted into solar irradiance or used as a calibrated measure of environmental light conditions.

**Figure 5:**
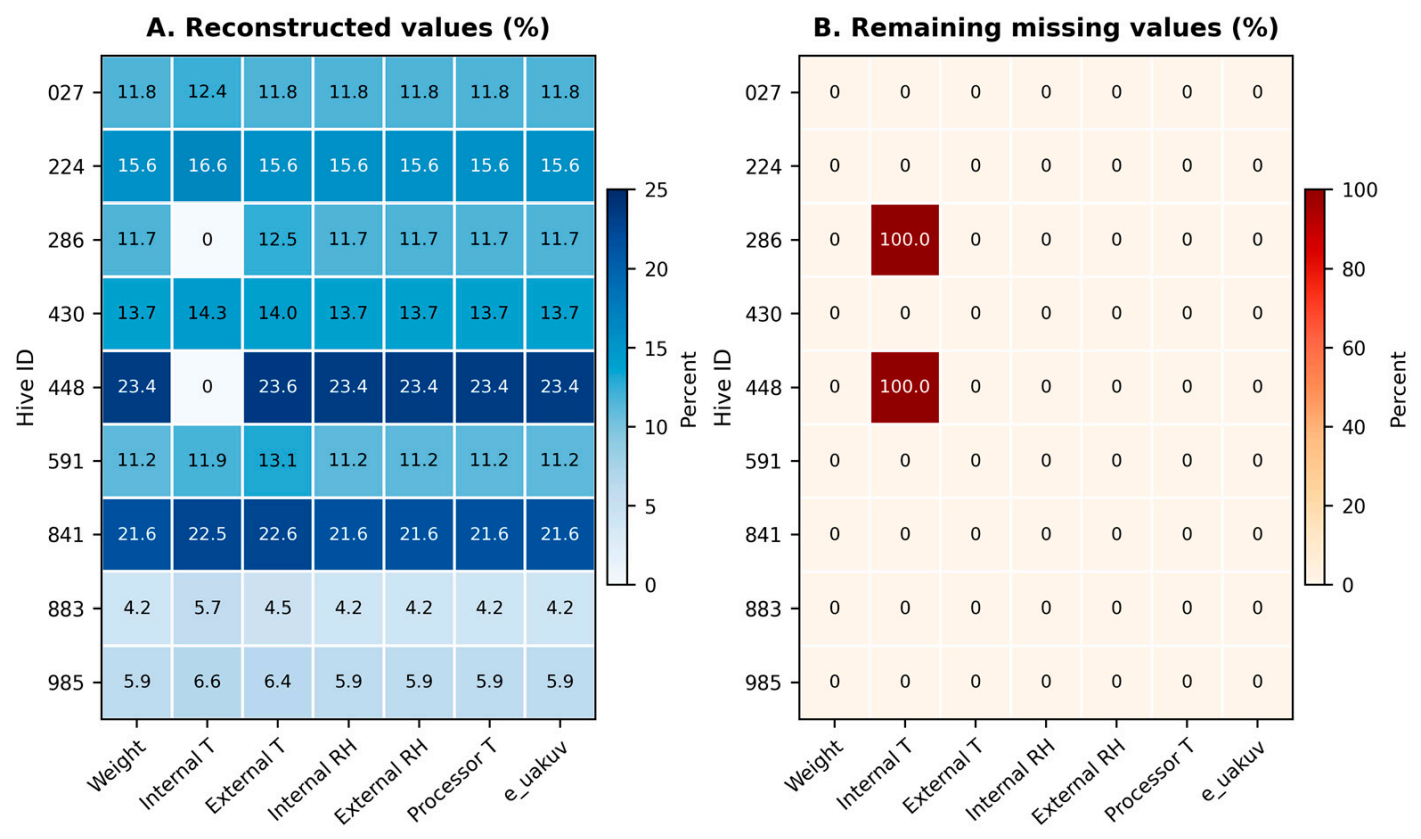
Percentages of reconstructed and remaining missing hourly values by hive and telemetry channel in the released CLEAN layer. Percentages are calculated separately within each hive file. Reconstructed values were identified from the channel-specific provenance fields; remaining missing values are empty after application of the reference workflow.

The distribution across individual nodes and channels is shown in Figure 5. Reconstruction percentages primarily reflect node-level record loss, whereas the remaining missing values are confined to the internal-temperature channel of hives 286 and 448.

The 5899 remaining internal-temperature values correspond entirely to hives 286 and 448. In their RAW files, temp1 returned 0 °C throughout the deployment (2606 and 2257 records, respectively); under the predefined quality-control rule, these values were treated as technical failures. Because neither channel contained valid observations, interpolation or a hive-specific diurnal profile could not be estimated, and the workflow deliberately left the full series missing. Internal-temperature analyses are therefore limited to the other seven hives.

### 4.3. Consistency of Co-Located External Sensors

Before relocation, the 36 pairwise external-temperature comparisons had a median correlation of 0.937 (pair-specific range: 0.908–0.998) and a median absolute pairwise difference of 1.0 °C (range of pair-specific medians: 0.3–2.3 °C). The number of simultaneous directly observed values per pair ranged from 961 to 1540. During the pollination campaign, the 12 comparisons within the three co-located groups produced a median correlation of 0.967 (0.930–0.995) and a median absolute difference of 1.3 °C (0.4–2.4 °C), based on 208–385 paired observations.

Agreement was weaker for external relative humidity. Before relocation, the median pairwise correlation was 0.771 (0.095–0.950) and the median absolute difference was 13.1 percentage points (range of pair-specific medians: 0.0–52.8), based on 987–1554 paired observations. During the campaign, the corresponding values were 0.846 (0.541–0.943) and 3.4 percentage points (0.0–33.5), based on 208–389 observations. Thus, the parallel external-temperature channels tracked shared conditions consistently, whereas the humidity channels showed substantial unit-specific differences. The distributed sensors provide useful cross-checking and anomaly-detection capacity, but their readings should not be treated as interchangeable or as a substitute for a calibrated weather station.

### 4.4. Limited Quality Check of the CLEAN Layer

The limited mask-and-reconstruct check was applied to hive weight, the primary continuous variable used to characterize node-level data availability in this field deployment. Hive-weight MAE ranged from 0.143 to 0.294 kg and RMSE from 0.375 to 1.297 kg across the nine hives. These values provide a bounded technical check of the released CLEAN layer and are not assumed to apply to the temperature, humidity, processor-temperature, or solar-energy-proxy channels. Comparison across channels and with alternative reconstruction algorithms is reserved for a separate methodological study.

This experiment evaluates reconstruction of randomly masked individual hive-weight observations. It does not estimate reconstruction accuracy for consecutive empirical gaps, particularly the longest interruptions observed in the dataset.

### 4.5. Illustrative RAW-to-CLEAN Output

**Figure 6:**
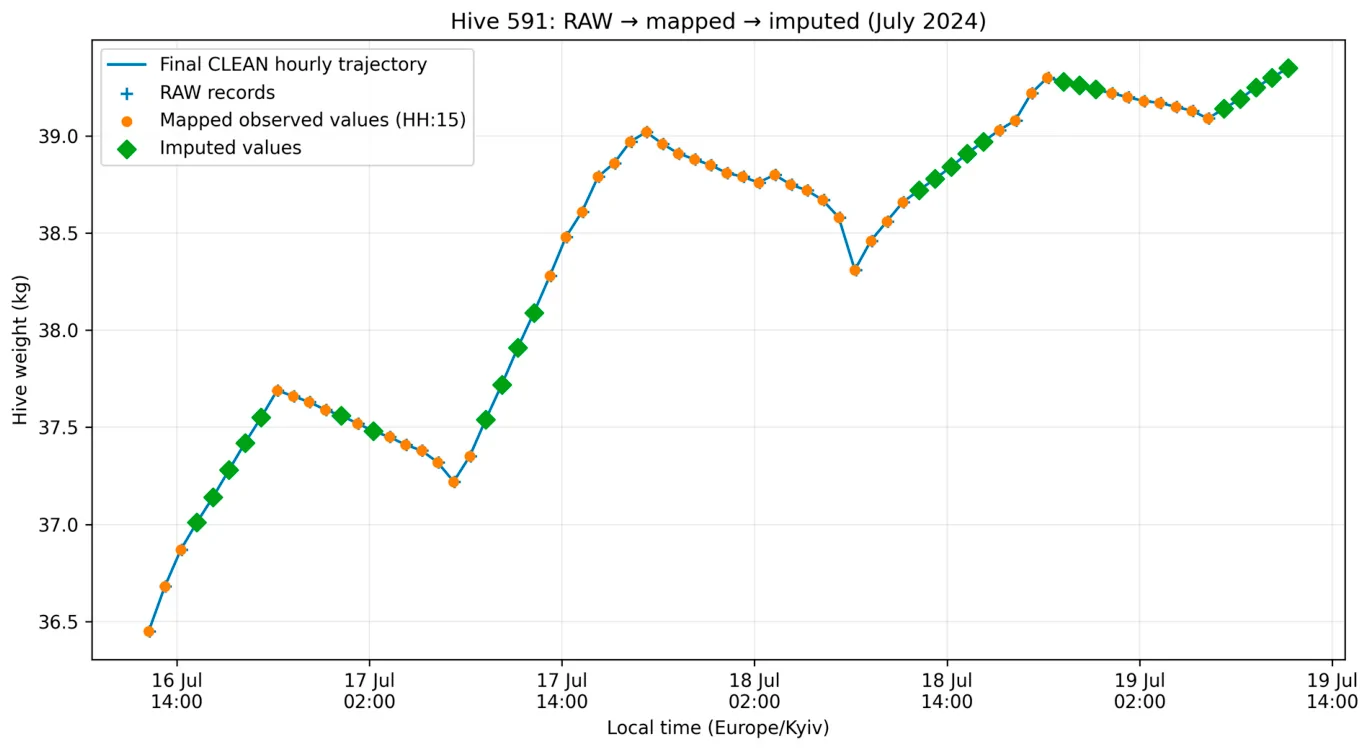
Illustrative transformation of irregular RAW hive-weight telemetry into the hourly CLEAN series for hive 591 in July 2024. Plus symbols denote RAW records, circles mapped observations, diamonds reconstructed values, and the line the final hourly trajectory.

Figure 6 shows an example of the mapping and reconstruction output for hive 591 in July 2024.

## 5. Discussion

The main contribution of this work is the combination of a decentralized autonomous smart-hive architecture, its use in a real commercial pollination deployment, and the resulting open multichannel dataset. Each hive operated as an independent solar-powered IoT node with its own sensors and GSM communication, allowing selected instrumented colonies to be relocated without dependence on a shared local concentrator.

The operational results put numerical bounds on this field performance. All nine nodes produced temporally alignable records, although coverage differed by 19.2 percentage points between the least and most complete streams. Most interruptions lasted only one or two hours. Decentralization therefore did not eliminate node-level data gaps, but it prevented a shared gateway from becoming an additional local dependency and made the quality of each node directly auditable. The present results characterize overall field operation; they do not isolate the effect of the relocation interval itself.

The decentralized and gateway-based topologies therefore involve different engineering trade-offs rather than a general ranking. In the AmoHive topology, loss of one node’s power supply, electronics, or cellular connection affects that node but does not interrupt the remaining hives, whereas a shared gateway creates a common local dependency. The price of this isolation is duplicated hardware, multiple cellular radios, distributed battery and solar-power maintenance, and potentially one cellular subscription per hive. The hourly duty cycle limits radio activity and energy demand, but a gateway design can use lower-power short-range links at the hives and concentrate cellular transmission in one device. Adding an AmoHive node requires no reconfiguration of a local gateway and preserves portability when individual hives are moved; however, hardware, service, and maintenance costs increase approximately with the number of monitored hives. A gateway can reduce duplicated equipment and recurring communication costs in a fixed apiary, but its coverage, capacity, and placement must be reconsidered when the monitored set expands or is divided among distant locations. The appropriate topology therefore depends on deployment scale, cellular availability, relocation frequency, and the acceptable balance between common-mode and node-specific failure.

This study complements the previously published Data Note [23] and the associated Zenodo repository [24]. The Data Note introduced the campaign and dataset; here they are used to examine the monitoring architecture, operational deployment, data organization, and the relationship between the RAW and CLEAN layers.

The systems summarized in Table 2 occupy different communication-architecture design points. WBee routes XBee node traffic through a local server before cloud transfer [26]; the Cecchi et al. system uses hive-level Bee boards, a Queen board, and a wireless bridge [27]; and MSPB sends wireless sensor data to a solar-powered central aggregator [17]. These arrangements centralize part of the communication or storage chain. AmoHive instead duplicates sensing, power, timing, and GSM communication at every hive and removes the local aggregation layer. This supports relocation and node-level fault isolation, but at the cost of repeated hardware, distributed maintenance, and potentially one cellular subscription per monitored hive.

The compared systems also make different temporal trade-offs. Meikle et al. used 1 min records in targeted experiments and 15 min records in selected longitudinal datasets [11]; WBee and the system of Andrijević et al. used 5 min acquisition [26,29]; MSPB used 15 min records [17]; and the low-power system of Bratek and Dziurdzia recorded only three scheduled measurements per day [28]. AmoHive’s nominal hourly cycle lies between these approaches. It cannot resolve rapid sub-hourly transitions, but it reduces radio activity and communication demand while preserving within-day weight and microclimate structure across independently operating hives. The appropriate design therefore depends on whether temporal detail, energy autonomy, infrastructure simplicity, or relocation flexibility is the dominant requirement.

Low-duty-cycle operation also shortens the active time of electronics and GSM communication near the colony. This was an engineering choice; electromagnetic exposure and biological effects were not measured.

The timestamped event workbook, optionally embedded event fields, and study-level campaign metadata provide essential operational context. Hive inspections, relocations, equipment changes, and campaign interventions may generate abrupt telemetry changes that should not automatically be interpreted as colony-driven behavior. Linking these events to the hourly records permits downstream studies to exclude, stratify, or explicitly model such periods.

### 5.1. Dataset Reuse and Potential Applications

The separation of the RAW and CLEAN layers supports complementary forms of reuse. The RAW records can be used to examine timestamp behavior, communication interruptions, node-level availability, quality-control rules, and alternative alignment or reconstruction procedures. The hourly CLEAN series support cross-hive comparisons, seasonal weight and microclimate analyses, anomaly detection, sensor-consistency studies, and the development and testing of statistical or machine-learning models. Because provenance is retained for each reconstructed value, users can restrict an analysis to directly observed records or test its sensitivity to reconstruction. The timestamped event log can additionally be synchronized with the hourly records for analyses centered on inspection, equipment changes, and other documented interventions; relocation and campaign interventions are retained as study-level metadata.

### 5.2. Limitations

First, the dataset represents a single summer-season deployment in one regional and climatic context, and only four colonies were relocated to the two field sites. The campaign demonstrates operational feasibility but does not by itself support broad causal generalization regarding pollination treatments or regional colony responses.

Second, the dataset does not include independent agronomic endpoints such as sunflower yield or seed set, and precipitation and wind were not integrated as synchronized channels. The campaign and attractant application should therefore be treated here as operational metadata rather than as evidence of pollination efficacy.

Third, the nominal hourly interval represents broad day-scale and seasonal trajectories but cannot recover rapid sub-hourly behavioral or microclimatic transitions. No simultaneous high-frequency reference system was operated during the campaign, so the information loss associated with hourly acquisition was not directly quantified.

Fourth, the manufacturer specifications reported for the sensing components do not constitute a calibration or uncertainty assessment of the complete installed hive-monitoring system. No traceable reference weather station or full system-level calibration was operated during the deployment. Pairwise agreement among co-located external sensors therefore measures operational consistency rather than measurement accuracy; the observed relative-humidity differences also show that individual units should not be treated as interchangeable. The high frequency of 99.9% humidity outputs further limits absolute and cross-unit humidity comparisons. In addition, the complete loss of the internal-temperature channel in two nodes restricts analyses of that variable to seven hives.

Fifth, electromagnetic-field exposure generated by the monitoring hardware was not measured. Low-duty-cycle operation should therefore be interpreted as an energy-efficient and precautionary engineering principle, not as experimental evidence of reduced exposure or biological effect.

Finally, the mask-and-reconstruct check was limited to randomly selected individual hive-weight observations. Because isolated-point masking is less demanding than reconstruction of consecutive gaps, the reported MAE and RMSE values should not be extrapolated to empirical interruptions of different lengths. No equivalent reconstruction experiment was conducted for the other telemetry channels, and the hive-weight error values should not be transferred to those variables. The CLEAN layer depends on one documented reconstruction procedure that was not compared with alternative methods. A systematic evaluation using blocked masking, gap-length classes, and alternative reconstruction methods is beyond the scope of the present architecture and data-resource study. Analyses sensitive to reconstructed observations should use the supplied provenance fields.

## 6. Conclusions

We present a decentralized autonomous smart-hive IoT architecture and demonstrate its operation through season-long monitoring of nine structurally identical solar-powered hives and relocation of selected units during a commercial sunflower pollination campaign. All nodes produced records compatible with the common hourly grid; observed coverage ranged from 76.6% to 95.8%, and missing-data runs were predominantly one to two hours long. Each hive functioned as an independent sensing and GSM communication node, enabling apiary reconfiguration without a shared local concentrator while keeping node-level data availability transparent. These findings characterize the reported AmoHive implementation and deployment; they do not establish inherent superiority or greater robustness relative to gateway-based architectures.

The resulting open resource combines approximately hourly RAW telemetry, a reproducible CLEAN layer, value-level provenance, campaign metadata, and a timestamped beekeeper-event workbook with an embedding script. It connects the physical architecture and its field operation with a reusable multichannel dataset for subsequent precision-beekeeping studies.

## Figures and Tables

**Table 1:** Relationship between the present study and earlier AmoHive outputs, including the analytical approach used to extend each result.

Source	Previous Scope	How the Present Study Extends the Previous Result
[19]	Autonomous AmoHive unit and use of its telemetry for monitoring and forecasting honey-robbing events.	System-level evaluation of a deployment composed of nine independent AmoHive nodes, including temporal alignment, node-level availability, co-located sensor consistency, and field relocation.
[23]	Data Note describing the nine-hive pollination dataset, monitored variables, campaign structure, and released RAW and CLEAN records.	Architecture-level analysis of the released campaign data using node-specific coverage, missing-run statistics, channel completeness, and synchronized operational events.
[24]	Public repository containing the data, event metadata, documentation, provenance information, and processing materials.	Provenance-aware analysis of the paired RAW and CLEAN layers, including reconstructed-value summaries and a limited mask-and-reconstruct quality check.

**Table 2:** Temporal organization, data transmission, and analytical use in selected sensor-based honey bee colony-monitoring studies and systems.

Study/System	Non-Acoustic Sensor Channels Included	Acquisition or Logging Interval	Analytical Treatment/Resolution	Transmission and Storage Strategy	Main Use in the Source Study
Meikle et al. [11]	Hive weight	1 min in targeted experiments; 15 min in selected longitudinal datasets	Native within-day series analyzed using piecewise regression; daily breakpoint and segment-slope parameters were derived; a 25 h running average was additionally used for detrending in one field experiment	Electronic scales linked to local dataloggers; remote transmission was not described	Timing and magnitude of forager departure and return; colony foraging activity; nutritional and environmental effects
WBee, Gil-Lebrero et al. [26]	Hive weight; internal and ambient T and RH	Configurable from 5 to 15 min; 5 min in the sunflower-bloom field deployment	5 min primary records; daily averages were used in selected result visualizations	XBee nodes transmitted records to a local server, which forwarded them to the cloud; microSD storage was used during communication loss	Remote monitoring of hive weight and microclimate; field demonstration during sunflower bloom
Cecchi et al. [27]	Hive weight; internal and external T and RH; CO_2_; external weather variables	Weight values were read every 5 s; separate numerical logging intervals for T, RH, and CO_2_ were not reported	No common resampling interval was specified; examples examined 24-hour, 1-week, and 1-year measurement periods	Hive-level Bee boards transferred data to a Queen board; the Queen-board network communicated with a remote server through a 5 GHz wireless bridge; a distinct upload interval was not reported	Multisensor characterization of normal daily activity, honey gathering, and swarming events
Bratek and Dziurdzia [28]	Hive weight; outdoor T and RH; hive T; battery voltage	Three scheduled measurements per day	Three records per day; the cloud system supported daily visualization and longer-term monthly and annual comparisons	One GPRS upload was performed after the third daily measurement; every upload included records from the preceding 3 days	Energy-efficient remote hive weighing and environmental monitoring; evaluation of power demand and operating autonomy
Andrijević et al. [29]	Internal and external environmental variables; illumination and weather parameters; optical bee entrance/exit counts	5 min	Hourly averages for input variables; hourly sums for bee entrances and exits	Data were transferred through GPRS to a cloud/web database and were also written to local storage; a distinct upload interval was not reported	Hourly forecasting of bee entrance and exit activity; analytical and predictive alarm generation
MSPB, Zhu et al. [17]	Internal T and RH	15 min	15 min primary records; hourly aggregation was used to represent daily patterns and daily averaging to represent annual patterns	Wireless transmission to a solar-powered central aggregator followed by cloud storage; a distinct batch interval was not reported	Longitudinal dataset linking continuous sensor data with expert phenotypic assessments performed every two weeks; example analyses of temporal patterns and colony outcomes
AmoHive, present study (system architecture described in [19])	Hive weight; internal and external T and RH; processor T; solar-energy proxy	One nominal multichannel record approximately every 60 min; no higher-frequency local logging was used	Regularized 60 min CLEAN time series on the fixed HH:15 grid	One GSM transmission session per nominal acquisition cycle, followed by sleep mode	Season-long field telemetry; campaign metadata and optional synchronization with beekeeper events; quality control, imputation, provenance tracking, and future time-series modeling

Acoustic and vibration channels were excluded from the channel comparison. T, temperature; RH, relative humidity. Acquisition, logging, transmission, and analytical intervals are distinct system properties. The table presents representative primary studies rather than an exhaustive review.

**Table 3:** Main hardware components and source-reported operating specifications of the AmoHive monitoring unit.

Source-Reported Specification	Function and Placement	Model or Implementation	Component
Controls sensor power, battery-state logic, data acquisition, record formatting, and low-power operation	Central control of the acquisition-and-transmission cycle	ATmega328P	Microcontroller
Manufacturer specification [40]: temperature range −40 to 80 °C, accuracy <±0.5 °C, resolution 0.1 °C, repeatability ±0.2 °C; relative-humidity range 0–100% RH, accuracy ±2% RH (maximum ±5% RH), resolution 0.1% RH, repeatability ±1% RH	Internal sensor on the rear wall of the brood chamber; external sensor in the lower part of the sealed rear-wall IoT block	DHT22/AM2302; two identical units	Temperature and relative-humidity sensors
Manufacturer specification [41]: rated capacity 200 kg; OIML R60 accuracy class C3 (3000 verification intervals); rated output 2.0 ± 0.2 mV/V; ingress protection IP65	Single strain-gauge load cell integrated into the metal scale structure beneath the hive	L6E 200 kg C3, Zemic Europe B.V.	Hive-weight sensor
Activated during each scheduled acquisition-and-transmission cycle	Mobile-network registration, GPS acquisition, and telemetry transmission to the server	SIM5320 GSM/GPS module	Cellular and positioning module
Supports a nominal 1 h cycle above +5 °C and a 2 h cycle below +5 °C in the operating logic described in [19]	Cycle timing and wake-up control	DS3231	Real-time clock
Low-power mode below 12.0 V; normal operation resumes above 12.8 V	Energy buffering and solar charging	UL0.8-12JST battery; LM2576ADJ-based charging module	Battery and charging circuit
Rated power: 20 W; rated current: 1.15 A; rated voltage: 17.5 V; open-circuit voltage: 22.0 V; dimensions: 340 × 450 × 25 mm	Mounted vertically on the front wall of the hive	CL020-12, CEL-LINE	Solar panel

The specifications in Table 3 are manufacturer-reported values for individual components under their stated test conditions. They do not constitute an in situ calibration or an uncertainty budget for the assembled hive-scale and sensor installation. A traceable system-level uncertainty estimate was not available for this deployment; absolute comparisons among nodes should therefore be interpreted together with the empirical cross-node consistency results reported in Section 4.3.

**Table 4:** Operational telemetry summary for the nine autonomous AmoHive nodes. Coverage is calculated before reconstruction; gap statistics refer to consecutive missing hourly hive-weight records.

Hive	Observed (%)	Median |Δ| (min)	Max |Δ| (min)	Gap Runs (N)	Median Gap (h)	Max Gap (h)
027	88.2	1	6	218	1	12
224	84.4	12	15	249	1	17
286	88.3	15	18	240	1	7
430	86.3	1	6	240	1	10
448	76.6	1	30	226	2	9
591	88.8	3	9	195	1	7
841	78.4	9	12	304	1	8
883	95.8	26	30	74	1	5
985	94.1	25	30	86	1	7

**Table 5:** Pooled technical summary of the seven telemetry channels in the released CLEAN layer (nine hives; 26,559 hourly records).

Variable (Unit)	Directly Observed, n (%)	Reconstructed, n (%)	Remaining Missing, n (%)	Median	IQR	Range
Hive weight (kg)	23,047 (86.8)	3512 (13.2)	0 (0.0)	32.18	26.81–38.60	7.66–69.16
Internal temperature (°C)	18,006 (67.8)	2654 (10.0)	5899 (22.2)	27.0	24.2–29.9	5.0–40.7
External temperature (°C)	22,898 (86.2)	3661 (13.8)	0 (0.0)	21.1	17.0–26.3	1.3–46.5
Internal relative humidity (%)	23,047 (86.8)	3512 (13.2)	0 (0.0)	99.9	64.0–99.9	0.0–99.9
External relative humidity (%)	23,047 (86.8)	3512 (13.2)	0 (0.0)	99.9	62.0–99.9	0.0–99.9
Processor temperature (°C)	23,047 (86.8)	3512 (13.2)	0 (0.0)	22.25	17.75–28.25	0.25–52.25
Post-stabilizer voltage, e_uakuv (V)	23,047 (86.8)	3512 (13.2)	0 (0.0)	13.24	12.85–13.54	12.03–14.25

Directly observed and reconstructed counts were determined from the corresponding *_method provenance fields. Summary values include both observed and reconstructed non-missing records. Statistics are descriptive and pooled across hives, dates, locations, and campaign groups; they are not estimates of treatment effects.

## Data Availability

The dataset analyzed during this study is publicly available in Zenodo: Kurdin, I.; Kurdina, A. Time-series dataset of non-invasive IoT measurements in *Apis mellifera* colonies: sunflower pollination service during summer season (2024; Ukraine), Version 1.1, 2026. https://doi.org/10.5281/zenodo.17966409.

## Abbreviations

The following abbreviations are used in this manuscript:

IoT	Internet of Things
HH:15	Fixed hourly timestamp grid at minute 15
RAW	Raw telemetry layer/original device-export data
CLEAN	Cleaned hourly telemetry layer
